# Knowledge and attitude of women towards maternity waiting homes and associated factors among women who gave birth in the last twelve months in Dega Damot district, northwest Ethiopia

**DOI:** 10.3389/fgwh.2023.988328

**Published:** 2023-02-20

**Authors:** Tazeb Alemu Anteneh, Abayneh Aklilu Solomon, Animut Tagele Tamiru, Nebiyu Solomon Tibebu, Marta Yimam Abegaz, Wubedle Zelalem Temesgan, Azmeraw Ambachew Kebede, Mastewal Belayneh Aklil, Tiruye Tilahun Mesele, Tiringo Molla Tiruye

**Affiliations:** ^1^ Department of Clinical Midwifery, School of Midwifery, College of Medicine and Health Sciences, University of Gondar, Gondar, Ethiopia; ^2^ Department of General Midwifery, School of Midwifery, College of Medicine and Health Sciences, University of Gondar, Gondar, Ethiopia

**Keywords:** attitude, Ethiopia, knowledge, maternity waiting homes, factors

## Abstract

**Background:**

Maternal waiting homes have been identified as one strategy to reduce maternal and perinatal mortality by bringing women living in hard-to-reach areas closer to a health facility that provides emergency obstetric care. Even if utilization of maternal waiting homes is repeatedly assessed, there is a scarcity of evidence in Ethiopia regarding women's knowledge and attitude towards maternal waiting homes.

**Objective:**

This study aimed to assess women's knowledge and attitude towards maternity waiting homes and associated factors among women who gave birth in the last twelve months in northwest Ethiopia.

**Methods:**

A community-based cross-sectional study was conducted from January 1st to February 30th, 2021. A total of 872 participants were selected by using a stratified cluster sampling technique. Data were collected by face-to-face interviews using a structured, pretested, and interviewer-administered questionnaire. Data were entered into EPI data version 4.6 and analysis was done through SPSS version 25. The multivariable logistic regression model was fitted and the level of significance was declared at a *p*-value of ≤0.05.

**Result:**

Women's adequate knowledge and positive attitude towards maternal waiting homes were 67.3% (95% CI: 64–70) and 73% (95% CI: 70–76), respectively. Had antenatal care visit, the shortest distance to reach the nearby health facility, had history of maternal waiting homes utilization, always involved in health care decision, and sometimes involved in health care decisions were significantly associated with women's knowledge regarding maternal waiting homes. Moreover, secondary and above educational level of women, short distance to reach the nearby health facility and had antenatal care visit were significantly associated with women's attitude towards maternity waiting homes.

**Conclusion:**

About two-third of women had adequate knowledge and nearly three-fourth of women had a positive attitude towards maternity waiting homes. It is better to improve the accessibility and utilization of maternal health services Furthermore, it is better to promote women's decision making power and create motivation to have better academic achievement of women.

## 1. Introduction

A Maternity Waiting Homes (MWHs) are a facility, within easy reach of a hospital or health center where a pregnant woman can stay starting from the term period of pregnancy and await labor (1). The maternity waiting homes provide a setting where women living in hard-to-reach areas can be accommodated as they get to term near a hospital with essential obstetric facilities (2). The first MWHs in Ethiopia were established as early as 1973, at Attat Our Lady of Lourdes Catholic Primary Hospital (3). The MWHs are important to improve institutional delivery and facilitate reduction in maternal and neonatal mortality and improve maternal and neonatal outcomes by fast-tracking women to emergency care (3–5).

According to World Health Organization report, 830 women daily and 303,000 women annually die from complications related to pregnancy and childbirth globally (6). Developing regions accounted for approximately 99% (302,000) of the estimated global maternal deaths, and Sub-Saharan Africa alone roughly accounted for 66% (201,000) (6). In Ethiopia, 412 women die per 100,000 live births from pregnancy-related complications (7). Many of the maternal deaths have been attributed to barriers to access like unfavorable topography, long travel times, lack of transport, and the reluctance of women to move and seek care before the start of labor (8–10). One of the strategies to increase access to care and reduction of maternal morbidity and mortality is the improvement of MWHs utilization (11). But MWHs utilization is still low (12).

Lack of awareness and poor attitude of women are the main attributes of low utilization of MWHs (4, 13, 14). Some women believed that MWHs are reserved for weak women only and every woman admitted to MWHs will be operated. The presence of such misconception shows that women didn't understand the aim of MWHs and reduced their utilization (15). Studies done in Indonesia show that the knowledge and attitude of women towards MWHs were 41.9% and 39.5% respectively (16). Similarly, a study conducted in Kenya show that the knowledge and attitude of women towards MWHs were 61.1% and 33.6% respectively (17). Some of the factors associated with women's knowledge and attitude towards MWHs are women's educational level (16, 17), women's occupational status, husband's educational status (16), history of MWHs utilization, accessibility of MWHs, and counseled about MWHs during antenatal care visit (17).

Nowadays, maternal and neonatal mortality reduction is a global agenda as specified on the third component of sustainable development goal (SDG) (18). This could be realized through the improvement of MWHs utilization (1). Similarly, making motherhood safer is a priority for the Ethiopian government and other non-governmental partners by using different strategies including establishing the health extension program which has been directed to reduce maternal mortality and morbidity (19). But MWHs utilization is low and evidence reaching out to the knowledge and attitude of women regarding MWHs is scarce. Therefore, this study assessed the knowledge and attitude of women regarding MWHs and associated factors among women who gave birth in the last twelve months in Dega Damot district and it could be important for policymakers to act based on the finding. As a result, MWH utilization will be improved and our global and national goal on maternal and neonatal mortality reduction will succeed.

## 2. Methods

### 2.1. Study design, period, and setting

A community-based cross-sectional study was carried out from January 1st to February 30th, 2021, in Dega Damot district, northwest Ethiopia. The district's administrative town, Feres Bet, is located about 400 km northwest of Addis Ababa (the capital city of Ethiopia) and 117 km from Bahir Dar (the capital city of Amhara regional state). The district is administratively divided into 34 kebeles (the smallest administrative unit in Ethiopia), which are 2 urban and 32 rural kebeles. In addition, the district has one primary hospital, eight health centers, and thirty-two health posts. The overall antenatal care service utilization and institutional delivery in the district were 66% (20) and 38.2% (21) respectively.

### 2.2. Study population

All mothers who gave birth in the last twelve months and live in the selected clusters of Dega Damot district during the data collection period were included. Mothers who were seriously ill and unable to respond throughout the data collection period were excluded.

### 2.3. Sample size determination and sampling procedure

The sample size for this study was determined by using a single population proportion formula by considering the following assumptions: 50% proportion of knowledge and attitude towards MWHs (since there were no similar studies in Ethiopia), 95% level of confidence, and 5% margin of error.

$$=\frac{\;{\left(Z\alpha /2\right)}^{2}p(1-p)}{d^{2}}=\frac{{\left(1.96\right)}^{2}[0.5(1-0.5)]}{(0.05)2}=384$$

where *n *= required sample size, *z* = standard normal distribution curve value for 95% confidence level = 1.96, *α* = level of significance, *p* = proportion of knowledge and attitude on MWHs, and *d* = margin of error. By considering a design effect of 2 and a 10% non-response rate, the final sample size was 847. Dega Damot district has a total of 34 kebeles (2 urban and 32 rural). The kebeles were stratified into urban and rural. Nine kebeles (1 urban and 8 rural) were selected randomly. A house-to-house visit was conducted and all eligible women in the selected kebeles (clusters) were interviewed. A stratified cluster sampling technique was used to draw the final sample size. Finally, due to the effect of cluster sampling, a total of 872 women were interviewed.

### 2.4. Measurement and operational definitions

#### 2.4.1. Adequate knowledge

Five questions were prepared to assess knowledge of women about MWHs. For yes or no question, a participant who had respond “yes” were coded into 1 and who had respond “no” were coded into 0 and for other questions with more than one possible answer, at least one correct answer was coded as 1. The maximum and minimum scores were 5 and 0 respectively. Thus, based on the variables set to assess knowledge of women about MWHs, women who scored 60% and above were considered as having adequate knowledge about MWHs (22).

#### 2.4.2. Positive attitude

Eight questions were set to assess the attitude of women towards MWHs. Each question has a five-point Likert scale (1 = strongly disagree, 2 = disagree, 3 = neutral, 4 = agree, 5 = strongly agree). The total score ranged from 8 to 40. Thus, based on the variables set to assess the attitude of women towards MWHs, women who scored 60% and above were considered as having a positive attitude towards MWHs (22).

#### 2.4.3. Media exposure

Those who responded at least once a week for at least one of the media types (television, radio, or magazine) are considered to be regularly exposed (7).

### 2.5. Data collection tools, procedure, and quality control

The data collection tool was developed by reviewing the literature. A structured, interviewer-administered questionnaire was used to collect the data through face-to-face interviews. Initially, the questionnaire was prepared in English and translated to the local (Amharic) language, and back to English to ensure consistency. The questionnaire covers socio-demographic characteristics, reproductive and maternity healthcare characteristics, and knowledge and attitude-related questions. The study tool was assessed by senior researchers to evaluate and enhance the items in the question. Four diploma and two BSc midwives were recruited for data collection and supervision, respectively. A pretest was done on 5% (43) of the calculated sample size outside of the study area. One-day of training was given for data collectors and supervisors to assure language clarity and to give information on interview techniques, and how to keep the information. During the actual data collection period, the questionnaire was checked for completeness daily by the supervisors.

### 2.6. Data processing and analysis

The coded data were entered to EPI data version 4.6 and it was then exported to SPSS version 25 for cleaning and analysis purposes. The family wealth status was analyzed by using principal component analysis (PCA). Descriptive statistics were used to present the characteristics of the study participants. The binary logistic regression model was fitted to identify the factors associated with maternal knowledge and attitude towards MWHs. Variables having a *p*-value of ≤0.25 in the bivariable analysis were entered into a multivariable logistic regression analysis to identify independent factors associated with the knowledge and attitude of women towards MWHs. The multicollinearity assumption was assessed using the variance inflation factor (VIF) and the largest VIF was 5.2. To conduct a multivariable logistic regression analysis, variables were selected with the Backward Likelihood Ratio approach and model fitness was checked by using Hosmer Lemeshow test, a *p*-value of ≤0.05 with a 95% CI for the adjusted odds ratio was employed to ascertain the significant association.

### 2.7. Ethical considerations

The ethical approval letter was obtained from the School of Midwifery, on behalf of the Institutional Review Board (IRB) of the University of Gondar. A formal letter of organizational approval was obtained from Dega Damot district health office. Afterward, the information regarding the purpose of the study and the rights of the participants was provided to the study participants. Finally, written informed consent was obtained from each participant before the actual data collection.

## 3. Result

### 3.1. Sociodemographic related characteristics

A total of 872 women were interviewed in the current study. Of them, 853 participants had complete data giving a response rate of 97.8%. The median age of the participants was 31 years with an interquartile range of (27–36) and 51.6% of the participants were within the age group of ≥31 years old. Majorities (88%) of the respondents were married and 85.1% were rural dwellers. Regarding the husband's educational status slightly less than half (47.4%) of husbands had no formal education and 78.6% were farmers (Table 1).

**Table 1:** Sociodemographic characteristics of study participants in Dega Damot district, northwest Ethiopia, 2021 (*n* = 853).

Variables	Category	Frequency	Percentage (%)
**Age group of mothers in year**	≤20	12	1.4
	21–30	401	47.0
	≥31	440	51.6
**Wealth Quintile**	Lowest	169	19.9
	Second	172	20.2
	Middle	171	20.0
	Fourth	170	19.9
	Highest	171	20.0
**Residence**	Rural	726	85.1
	Urban	127	14.9
**Religion**	Orthodox	847	99.3
	Others^a^	6	0.7
**Educational status of women**	No formal education	572	67.1
	Primary (1–8)	135	15.8
	Secondary and above	146	17.1
**Occupational status of women**	Farmer	687	80.5
	Housewife	47	5.5
	Government employee	65	7.6
	Merchant	42	4.9
	Others^b^	12	1.4
**Current marital status**	Married	751	88.0
	Unmarried	102	12.0
**Husbands' occupational status (*n* = 751)**	Farmer	590	78.6
	Government employee	94	12.5
	Merchant	67	8.9
**Husband's educational status (*n* = 751)**	No formal education	356	47.4
	Primary (1–8)	206	27.4
	Secondary and above	189	25.2
**Traveling time to reach the nearby health facility**	≤30 min	314	36.8
	>30 min	539	63.2

^a^ Muslim and protestant.

^b^ student and private employee.

### 3.2. Reproductive and maternal health service-related characteristics

Nearly three-fourths (73.0%) of the study participants were multiparous. Slightly more than two-thirds (68.5%) of mothers had at least one ANC visit and nearly half (51.2%) of the respondents had a history of MWHs utilization (Table 2).

**Table 2:** Reproductive and maternal health service-related characteristics of study participants in Dega Damot district, northwest Ethiopia, 2021 (*n* = 853).

Variables	Category	Frequency	Percentage (%)
**History of MWHs utilization**	Yes	438	51.3
	No	415	48.7
**Parity**	Primiparous	230	27.0
	Multiparous	623	73.0
**ANC services utilization**	Yes	584	68.5
	No	269	31.5
**Number of ANC visit (*n* = 584)**	1–3	332	56.9
	≥4	252	43.1
**Health care related decision involvement**	Never	198	23.2
	Sometimes	54	6.3
	Always	601	70.5

### 3.3. Knowledge of women regarding maternity waiting homes

In the current study, 67.3% (95% CI: 64–70) of the participants had adequate knowledge about MWHs. About 69.2% of participants knew that women living in hard-to-reach areas have to visit the MWHs. In addition, about 63% of the study subjects identified that MWHs utilization could reduce maternal mortality and 80.3% of the participant knew that MWHs utilization reduces neonatal death (Table 3).

**Table 3:** Knowledge of women regarding MWH services in Dega Damot district, northwest Ethiopia, 2021 (*n* = 853).

No	Variables	Category	Frequency	Percentage (%)
1	Have you ever heard about MWH?	Yes	671	78.7
		No	182	21.3
2	What are benefits of MWH for mothers?	Reduce maternal death	537	63.0
		Help to get postpartum care	109	12.8
		Help to get health-related information	236	27.7
		Prevent delay	451	52.9
3	Is MWH important for newborn?	Yes	613	91.4
		No	58	8.6
4	What are benefits for newborn?	Reduce neonatal death	492	80.3
		Help to get post natal care	435	71.0
		Help to get immunization	417	768.0
5	Which mothers are eligible to visit MWH?	Pregnant women with hard to reach the nearby health facility	207	30.8
		All pregnant mothers	464	69.2
6	When should they visit MWH?	After 37 weeks	335	49.9
		After 32 weeks	244	36.3
		After 28 weeks	92	13.8

### 3.4. The attitude of women towards maternity waiting homes

In this study, about 73% (95% CI: 70–76) of the study participants had a positive attitude towards MWH services. About 61.1% of the participants agreed that waiting for delivery at MWH prevents pregnant women from reaching the clinic late due to long distances and lack of transport. In addition, about 58.5% of the study subjects agreed that waiting at MWHs will help women find assistance from the nurses and midwives if they develop labor complications (Table 4).

**Table 4:** Attitude of women towards MWH services in Dega Damot district, northwest Ethiopia, 2021 (*n* = 853).

No	Variables	Strongly agree frequency (%)	Agree frequency (%)	Not sure frequency (%)	Disagree frequency (%)	Strongly disagree frequency (%)
1	MWHs are important for the health of mothers and infants	209 (24.5)	447 (52.4)	74 (8.7)	123 (14.4)	0
2	It is worthwhile for pregnant to stay in MWH	19 (2.2)	238 (27.9)	169 (19.8)	308 (36.1)	119 (14.0)
3	Going early to MWHs is wiser than waiting for delivery at home until labor.	203 (23.8)	425 (49.8)	34 (4.0)	2 (0.2)	189 (22.2)
4	MWHs prevents pregnant due to long distances and lack of transport	88 (10.3)	521 (61.1)	2 (0.2)	169 (19.8)	73 (8.6)
5	Separation of women from families to stay at MWH does not hurt them.	103 (12.1)	260 (30.5)	113 (13.2)	375 (44.0)	2 (0.2)
6	MWH will help women find assistance from the nurses and midwives	132 (15.5)	499 (58.5)	34 (4.0)	1 (0.1)	187 (21.9)
7	A clinic with a MWH is more beneficial to the mother and baby than a clinic without MWH	187 (21.9)	400 (46.9)	116 (13.6)	148 (17.4)	2 (0.2)
8	Staying in the MWH while waiting for delivery will safe guard mothers.	110 (12.9)	556 (65.2)	4 (0.5)	183 (21.5)	0

### 3.5. Factors associated with women's knowledge regarding maternity waiting homes

On the logistic regression analysis, had ANC visit during the most recent pregnancy, short travel time to reach the nearby health facility, had health care decision involvement and history of MWHs utilization were the factors significantly associated with women's knowledge regarding MWHs.

In the current study, the odds of adequate knowledge regarding MWHs was 5.13 times higher among women who had ANC visits during the most recent pregnancy as compared to those women who had no ANC visits (AOR = 5.13; 95% CI: 3.60–7.30).

Similarly, knowledge of women regarding MWHs was 4.76 times higher among women who travel 30 min and less to reach the nearby health facility as compared to women who travel more than 30 min (AOR = 4.76; 95% CI: 3.09–7.31).

In addition, the odds of adequate knowledge regarding MWHs was 2.22 times more likely among women who had sometimes been involved in healthcare decisions as compared to women who had never been involved in healthcare decisions (AOR = 2.22; 95% CI: 1.01–4.92). Likewise, the odds of adequate knowledge regarding MWHs was 1.56 times higher among women who had always been involved in healthcare decisions as compared to women who had never been involved in healthcare decisions (AOR = 1.56; 95% CI: 1.05–2.32).

Lastly, a history of MWHs utilization increases women's knowledge by 2.13 times as compared to those women who had no a history of MWHs utilization (AOR = 2.13; 95% CI: 1.48–3.05) (Table 5).

**Table 5:** Multivariable logistic regression analysis of factors associated with knowledge about MWH services in Dega Damot district, northwest Ethiopia, 2021.

Variables	Category	Knowledge		COR (95% CI)	AOR (95% CI)
		Adequate	Inadequate		
**Women's educational status**	No formal education	336	236	1	1
	Primary	108	27	2.81 (1.79, 4.42)	1.33 (0.77, 2.29)
	Secondary and above	130	16	5.71 (3.31, 9.85)	1.44 (0.75, 2.78)
**Residence**	Rural	466	260	1	1
	Urban	108	19	3.17 (1.90, 5.29)	0.72 (0.37, 1.41)
**ANC utilization**	Yes	471	113	6.72 (4.88, 9.25)	5.13 (3.60, 7.30)*
	No	103	166	1	1
**Traveling time to reach the nearby health facility**	≤30 min	281	33	7.15 (4.80, 10.65)	4.76 (3.09, 7.31)*
	>30 min to	293	246	1	1
**Parity**	Primiparous	201	29	4.65 (3.05, 7.07)	1.87 (0.80, 2.56)
	Multiparous	373	250	1	1
**Access to media**	No	351	198	1	1
	Yes	223	81	1.55 (1.14, 2.11)	1.08 (0.73, 1.60)
**Health care related decision involvement**	Never	111	87	1	1
	Sometimes	39	15	2.04 (1.06, 3.94)	2.22 (1.01, 4.92)*
	Always	424	177	1.88 (1.35, 2.61)	1.56 (1.05, 2.32)*
**History of MWH utilization**	Yes	323	115	1.84 (1.37, 2.45)	2.13 (1.48, 3.05)*
	No	251	164	1	1

AOR, adjusted odds ratio; COR, crude odds ratio; CI, confidence interval.

*p-value ≤ 0.001.

### 3.6. Factors associated with women's attitude towards maternity waiting homes

On the multivariable logistic regression analysis, had ANC visit during the most recent pregnancy, short travel time to reach the nearby health facility and women's educational level of secondary and above were the factors significantly associated with women's attitude towards MWHs.

In this study, the odds of a positive attitude towards MWHs was 1.83 times higher among women who had secondary and above educational attainment as compared to women who had no formal education (AOR = 1.83; 95% CI: 1.03–3.24).

Similarly, attitudes of women towards MWHs were 2.66 times higher among women who had ANC visits during the most recent pregnancy as compared to those women who had no ANC visits (AOR = 2.66; 95% CI: 1.99–3.71).

In addition, the odds of a positive attitude towards MWHs was 2.17 times higher among women who travel 30 min and less to reach the nearby health facility as compared to women who travel more than 30 min (AOR = 2.17; 95% CI: 1.45–3.25) (Table 6).

**Table 6:** Multivariable logistic regression analysis on factors associated with women attitude towards MWH services in dega damot district, northwest Ethiopia, 2021.

Variables	Category	Attitude		COR (95% CI)	AOR (95% CI)
		Positive	Negative		
**Women's educational status**	No formal education	382	190	1	1
	Primary	113	22	2.56 (1.53, 4.17)	1.12 (0.87, 1.98)
	Secondary and above	128	18	3.54 (2.10, 5.97)	1.83 (1.03, 3.24)*
**Residence**	Rural	514	212	1	1
	Urban	109	18	2.50 (1.48, 4.22)	0.89 (0.47, 1.69)
**ANC services utilization**	Yes	474	110	3.47 (2.53, 4.77)	2.66 (1.99, 3.71)*
	No	149	120	1	1
**Traveling time to reach the nearby health facility**	≤30 min	271	43	3.35 (2.32, 4.83)	2.17 (1.45, 3.25)*
	>30 min to	352	187	1	1
**Parity**	Primiparity	192	38	2.25 (1.53, 3.32)	1.17 (0.74, 1.87)
	Multiparty	431	192	1	1
**Access to media**	No	384	165	1	1
	Yes	239	65	1.58 (1.14, 2.20)	1.13 (0.79, 1.64)
**History of MWH utilization**	Yes	336	102	1.47 (1.08, 1.99)	1.12 (0.83, 1.31)
	No	287	128	1	1
**Wealth status**	Lowest	112	57	1	1
	Second	115	57	1.03 (0.66, 1.61)	1.05 (0.65, 1.70)
	Middle	150	41	1.61 (1.00, 2.59)	1.54 (0.93, 2.56)
	Fourth	129	41	1.60 (0.99, 2.57)	1.53 (0.92, 2.63)
	Highest	137	34	2.05 (1.25, 3.36)	1.56 (0.92, 2.63)

AOR, adjusted odds ratio; COR, crude odds ratio; CI, confidence interval.

*p-value ≤ 0.001.

## 4. Discussion

Maternity waiting homes have been identified as one strategy to reduce maternal and perinatal mortality. Therefore, this study assessed the knowledge and attitude of women towards MWHs and associated factors in Dega Damot district, northwest Ethiopia. As a result, this study indicated that two-third of women had adequate knowledge and nearly three-fourth of them had a positive attitude towards MWHs. Short travel time to reach the nearby health facility, history of MWHs utilization, had ANC visit, and women involvement in health care-related decision were factors significantly associated with the women's knowledge about MWHs. In addition, higher women's educational level had ANC visits, and short traveling time were factors significantly associated with women's attitude towards MWHs.

In this study, about 67.3% of women had adequate knowledge regarding MWHs. This is higher than studies done in Kenya (33.6%) (17) and Indonesia (41.9%) (16). The variation might be due to sociodemographic difference in participants. From study done in Kenya, majorities (83.7%) of participants were housewives while only 5.5% were housewives in the current study. Housewives have less decision-making power in health care and other household activities (23). Evidence show that women's involvement in health care-related decisions was an important factor in MWHs utilization (24). In turn, it could have a significant effect on the knowledge of women about MWHs.

Similarly, about 73.0% of women had a positive attitude towards MWHs. This is higher than studies done in Kenya (61.1%) (17) and Indonesia (39.5%) (16). The variation might be due to sociodemographic differences in study subjects. In a study done in Kenya, study participants were all reproductive-age women while participants in the current study were women who gave birth in the last twelve months. Evidence show that increased parity had an association with MWHs utilization as compared to primiparity (24). But a study conducted in Kenya included both parous and nulliparous reproductive-age group women which in turn affects women's attitude towards MWHs.

This study indicated that having ANC visit was significantly associated with women's knowledge regarding MWHs. Having ANC visits increases women's knowledge regarding MWHs by 5.13 times as compared to its counterpart. The possible explanation might be women who had ANC visits can discuss with health care providers about different health care activities and will have better information regarding danger signs and obstetrics complications that may occur during pregnancy and childbirth and also have awareness regarding birth preparedness and complication readiness (25). Nowadays, MWHs utilization is one part of birth preparedness and complication readiness plan and pregnant mothers can get information on when how they visit MWHs (26). In addition, women who had ANC visits could receive information regarding the benefit and means of accessing MWHs from the trusted source, become more intended to use the service and develop increased health literacy regarding MWHs (27).

The odds of adequate knowledge regarding MWHs was 4.76 times higher among mothers who travel thirty minutes and less to reach the nearby health facility as compared to mothers traveling more than thirty minutes. This might be because distance is the commonest barrier to use maternal and child health services (28). Those mothers who need short traveling time to reach the nearby health facility can easily access health-related information, could have a better health-seeking behavior, and use different maternity services which are important to discuss about the benefit of waiting homes (29). Therefore, they might have adequate knowledge regarding MWHs.

History of MWHs utilization is an important factor associated with knowledge of women regarding MWHs. The odds of adequate knowledge about MWHs are 2.13 times more likely among women who had a history of MWHs utilization as compared to their counterparts. Those women who had a history of MWHs utilization might have a better understanding of the benefit of staying in MWHs (30). This might increase women's knowledge regarding MWHs.

Similarly, the involvement of women in health care-related decision was significantly associated with women's knowledge regarding MWHs. The odds of adequate knowledge regarding MWHs was 2.22 times more likely among women sometimes involved in healthcare-related decisions and 1.56 times more likely among women always involved in healthcare decision as compared to women never involved in healthcare-related decisions. The possible justification might be in Ethiopia, the burden of nurturing children, taking care of household tasks, and preparing food for the family is left to women. On the other hand, the decision regarding the household income and healthcare-related tasks are given to the husbands (14). Evidence shows that women's involvement on health care related decisions has a positive effect on MWHs utilization (24). In turn, it increases the knowledge of women regarding MWHs.

Higher educational attainment of women was another important factor associated with women's attitude towards MWHs. The odds of a positive attitude towards MWHs was 1.68 time higher among women with an educational level of secondary and above as compared to women who had no formal education. This is supported by a study done in Indonesia (16). This could be due to education being the main track for women's employment, improved household economic status, and empowerment on decision making which increases access to health care-related information (31). In addition, women with better educational attainment will have good health literacy which in turn influences them to seek information and advice from healthcare providers and will have a better view of the modern health care system (32). Therefore, they might have a positive attitude towards MWHs.

Short travel time to reach the nearby health facility was significantly associated with women's attitude towards MWHs. The odds of a positive attitude towards MWHs was 2.17 times more likely among women who had traveled thirty minutes or less to reach the nearby health facility as compared to those women who had traveled more than thirty minutes. The possible explanation might be the distance from the nearby health facility affects access to health care services and other information which is imperative for good health care-seeking practices (33). Women with short traveling time from the nearby health facility may frequently use maternal and child health services and may have better insight into its benefit (30). So, they could have a positive attitude towards MWHs.

Lastly, ANC visit was another important factor associated with women's attitude towards MWHs. The odds of a positive attitude towards MWH services was 2.66 times higher among women who had ANC visits as compared to those women who had no ANC visits. This is due to mothers who had ANC visits might be counseled by health professionals regarding the benefits of the MWHs and planning for the place of delivery (34, 35). In addition, women who had ANC visits might have the chance to familiarize themselves with the health facilities' environments which may reduce unnecessary fear and stress related to institutional services utilization (22). Therefore, they might have a positive attitude towards MWHs.

### 4.1. Limitation of the study

This study may have social desirability bias. To reduce social desirability bias, participants were informed in detail about the purpose of the study and ways of information handling to keep privacy. Despite these limitations, our findings provided important information about women's knowledge and attitude towards MWHs.

## 5. Conclusion

Two-thirds of women had adequate knowledge and nearly three-fourths of women had a positive attitude towards MWHs. Short travel time to reach the nearby health facility, had ANC service utilization, women educational status of secondary and above, women's involvement on health care-related decisions, and history of MWHs utilization were factors significantly associated with women's knowledge and attitude towards MWHs. So, stakeholders should work to improve the accessibility of maternal and child health services and better to arrange programs that will increase maternal and child health service utilization. Furthermore, it is better to promote women's decision-making power and better to create motivation for females to have better academic achievement.

## Data Availability

The original contributions presented in the study are included in the article/Supplementary Material, further inquiries can be directed to the corresponding author.
